# Supplementary material for: Phospho‐RNA‐seq: a modified small RNA‐seq method that reveals circulating mRNA and lncRNA fragments as potential biomarkers in human plasma
Source: EMBO J. 2019 May 3;38(11):e101695. doi: 10.15252/embj.2019101695 (PMC6545557; doi:10.15252/embj.2019101695)
Supplement: Supplementary file 1 — Appendix [file EMBJ-38-e101695-s001.pdf]

Appendix

Table of Contents

APPENDIX TABLES 1

Appendix Table S1. Demographic data for healthy control participants included in the study. 1

Appendix Table S2. Databases used for aligning contaminants. 2

Appendix Tables

Appendix Table S1. Demographic data for healthy control participants included in the study.

ID	Sex	Age
**ULMC 123**	Male	57
**ULMC 134**	Female	73
**ULMC 135**	Male	26
**ULMC 148**	Female	59
**ULMC 157**	Male	43

Appendix Table S2. Databases used for aligning contaminants.

Database ID	sRNAnalyzer Directory Path*
UniVec	UniVec/UniVec
human_miRNA	miRBase/hairpin_hsa_anno
human_miRNA_sub	miRBase/hairpin_hsa_sub_anno
human_piRNA	piRBase/piR_human_v1.0
human_snoRNA	snoRNABase/snoRNABase
virus_miRNA	miRBase/hairpin_virus_anno
plant_miRNA	miRBase/hairpin_plant_anno
all_miRNA	miRBase/hairpin_anno
all_miRNA_sub	miRBase/hairpin_sub_anno
human_endog_repMisc	UserDb/hg38_rmsk_miscRNA
bacteria_rRNA	RDP/release11_3_Bacteria_unaligned
archaea_rRNA	RDP/release11_3_Archaea_unaligned
fungi_rRNA	RDP/release11_3_Fungi_unaligned
all_rRNA_SSU	SILVA/SILVA_119_SSURef_tax_silva
all_rRNA_LSU	SILVA/SILVA_119_LSURef_tax_silva
human_repSeq	RepBase/humrep
human_subSeq	RepBase/humsub

This table is taken from the .config file used in running sRNAnalyzer. Runs were performed in “single” mode in the order presented in this table (top to bottom). All bowtie libraries, except “human_endog_repMisc”, are available from the sRNAnalyzer website, included in the “MainDBs” archive (http://srnanalyzer.systemsbiology.net/downloads/MainDBs.tar.gz).
*Directory paths are shown relative to the MainDBs directory created upon decompressing the MainDBs tarball. The “human_endog_repMisc” file was created by extracting genomic sequence (GRCh38) corresponding to RepeatMasker annotation tracks (UCSC; hg38) and gencode small RNA coordinates.
