# Supplementary material for: Phospho‐RNA‐seq: a modified small RNA‐seq method that reveals circulating mRNA and lncRNA fragments as potential biomarkers in human plasma
Source: EMBO J. 2019 May 3;38(11):e101695. doi: 10.15252/embj.2019101695 (PMC6545557; doi:10.15252/embj.2019101695)
Supplement: Supplementary file 2 — Expanded View Figures PDF [file EMBJ-38-e101695-s002.pdf]

Expanded View Figures

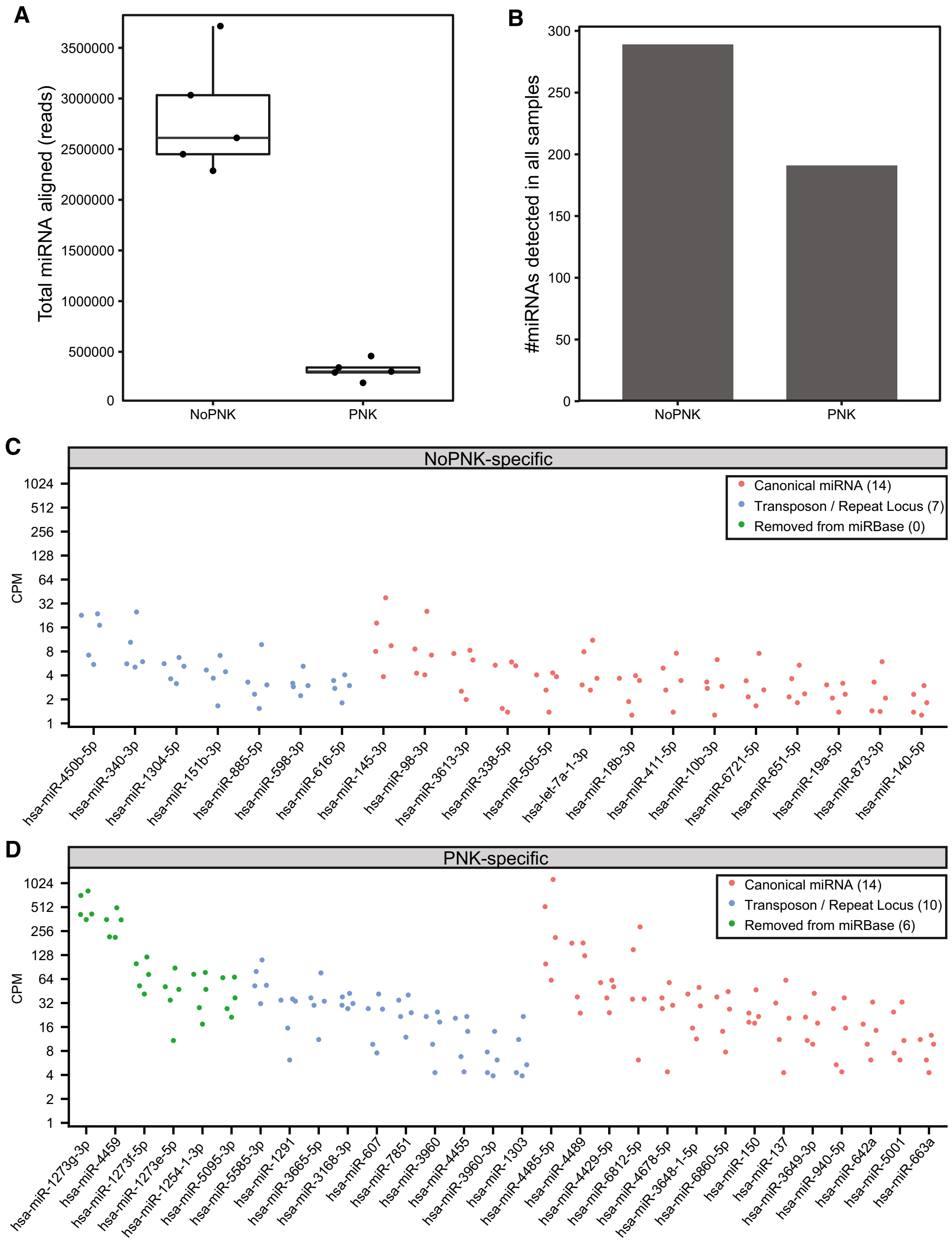

Figure EV1.

Figure EV1. Evaluation of extracellular microRNA recovery by phospho-RNA-seq and standard small RNA-seq.

A Boxplots summarize the total number of read alignments to human mature microRNAs. Overlaid points indicate the respective counts for five control individuals, obtained from three technical replicates. Boxes represent the mean ± interquartile range (IQR), and whiskers represent $1^{st}/3^{rd}$ quartile 1.5*IQR.

B Barplots indicate the number of mature microRNA genes detected (≥ 1 count) in all five individuals with and without PNK treatment.

C, D Dotplots show the expression level (counts per million) of microRNAs that were detected only in the (C) standard small RNA-seq or (D) phospho-RNA-seq protocols. Five points are shown for each miRNA, representing the miRNA abundance in each of the five controls. Green dots indicate microRNAs removed from the current version of miRBase (v22) due to lack of functional evidence. Blue dots indicate microRNAs overlapping transposable or repetitive element loci. Red dots represent microRNAs not overlapping transposons or repeats and which are present in the current version of miRBase. The number of microRNAs in each of the categories is shown in parentheses in the legend of the respective plots.

 © 2019 The Authors
